# Supplementary figures and images for: HCP Multi-Pipeline: a derived dataset to investigate analytical variability in fMRI
Source: Sci Data. 2025 Jun 4;12:940. doi: 10.1038/s41597-025-05247-7 (PMC12137627; doi:10.1038/s41597-025-05247-7)

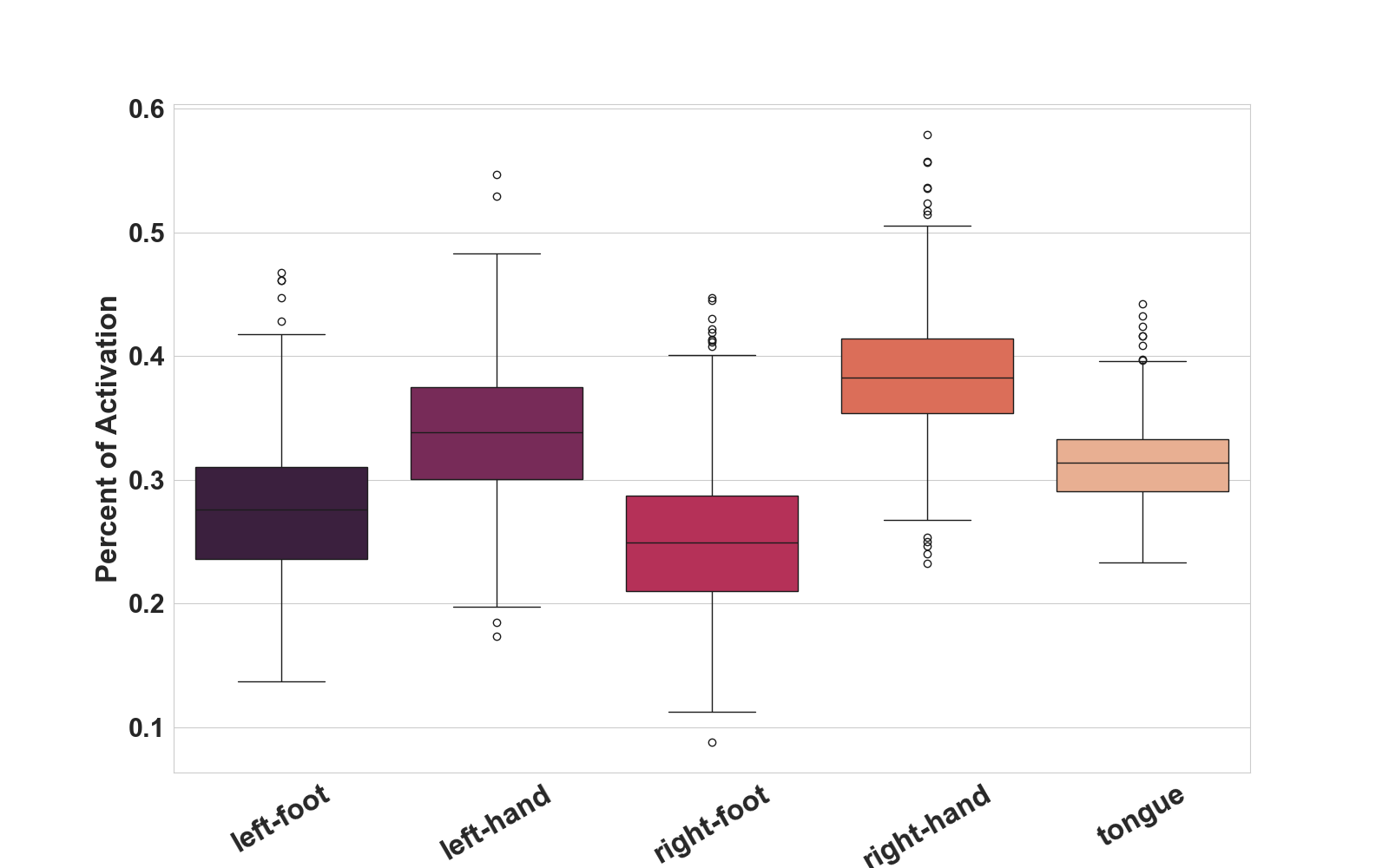

Supplement: Supplementary file 1 — Supplementary Materials [file 41597_2025_5247_MOESM1_ESM.zip › supplementary_materials/validation_FSL_5_6_0.png]

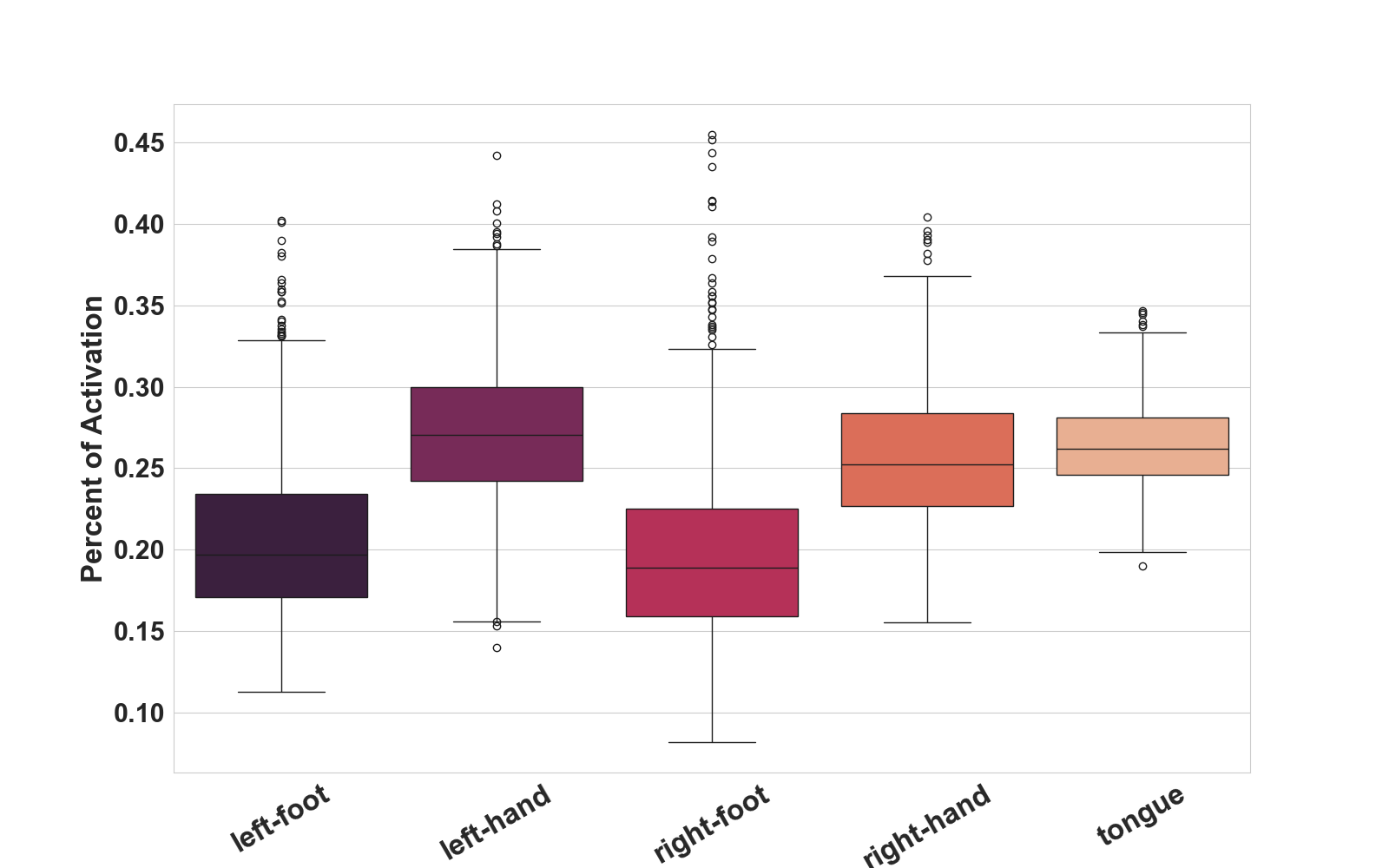

Supplement: Supplementary file 1 — Supplementary Materials [file 41597_2025_5247_MOESM1_ESM.zip › supplementary_materials/validation_FSL_5_6_1.png]

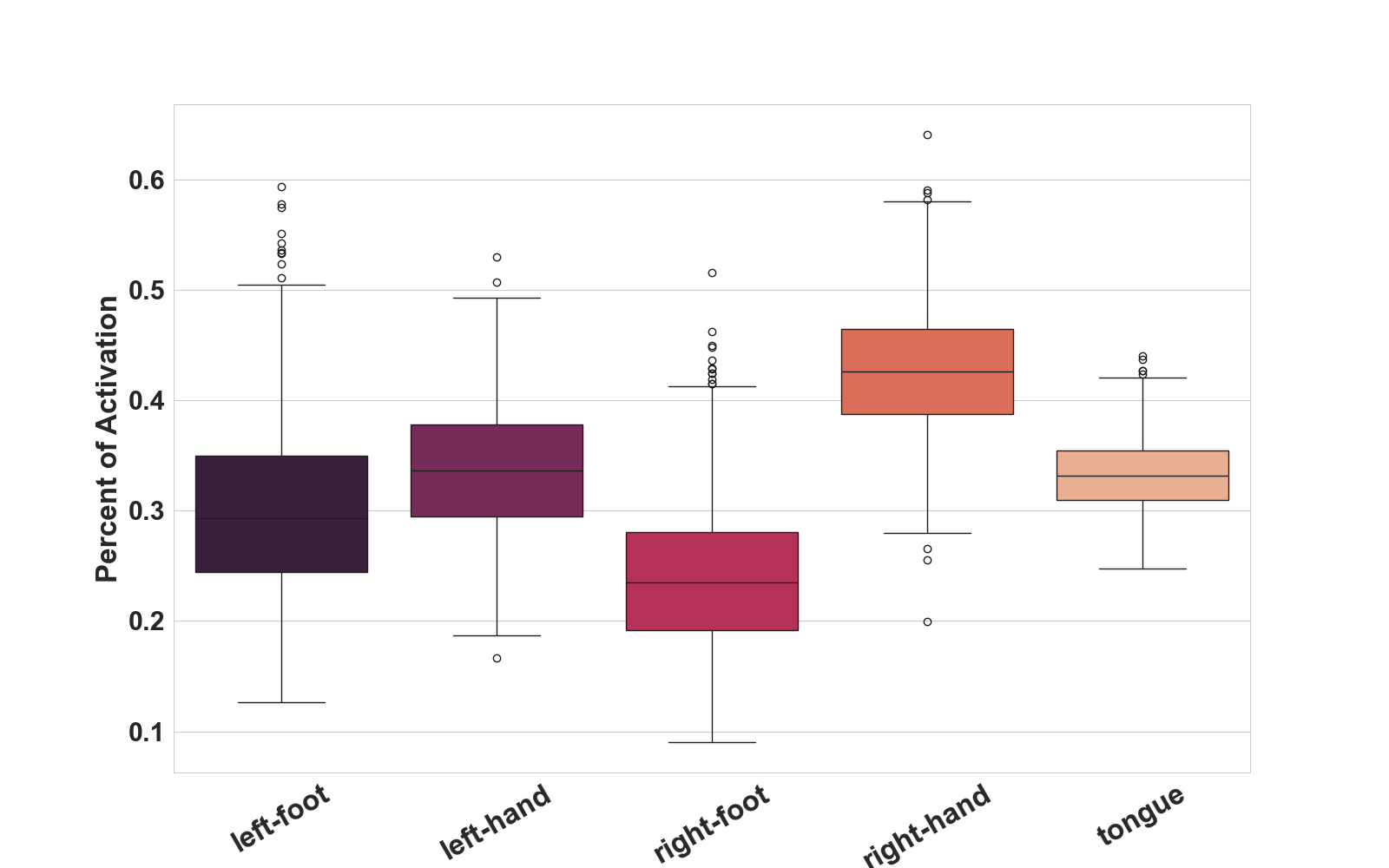

Supplement: Supplementary file 1 — Supplementary Materials [file 41597_2025_5247_MOESM1_ESM.zip › supplementary_materials/validation_SPM_8_6_0.png]

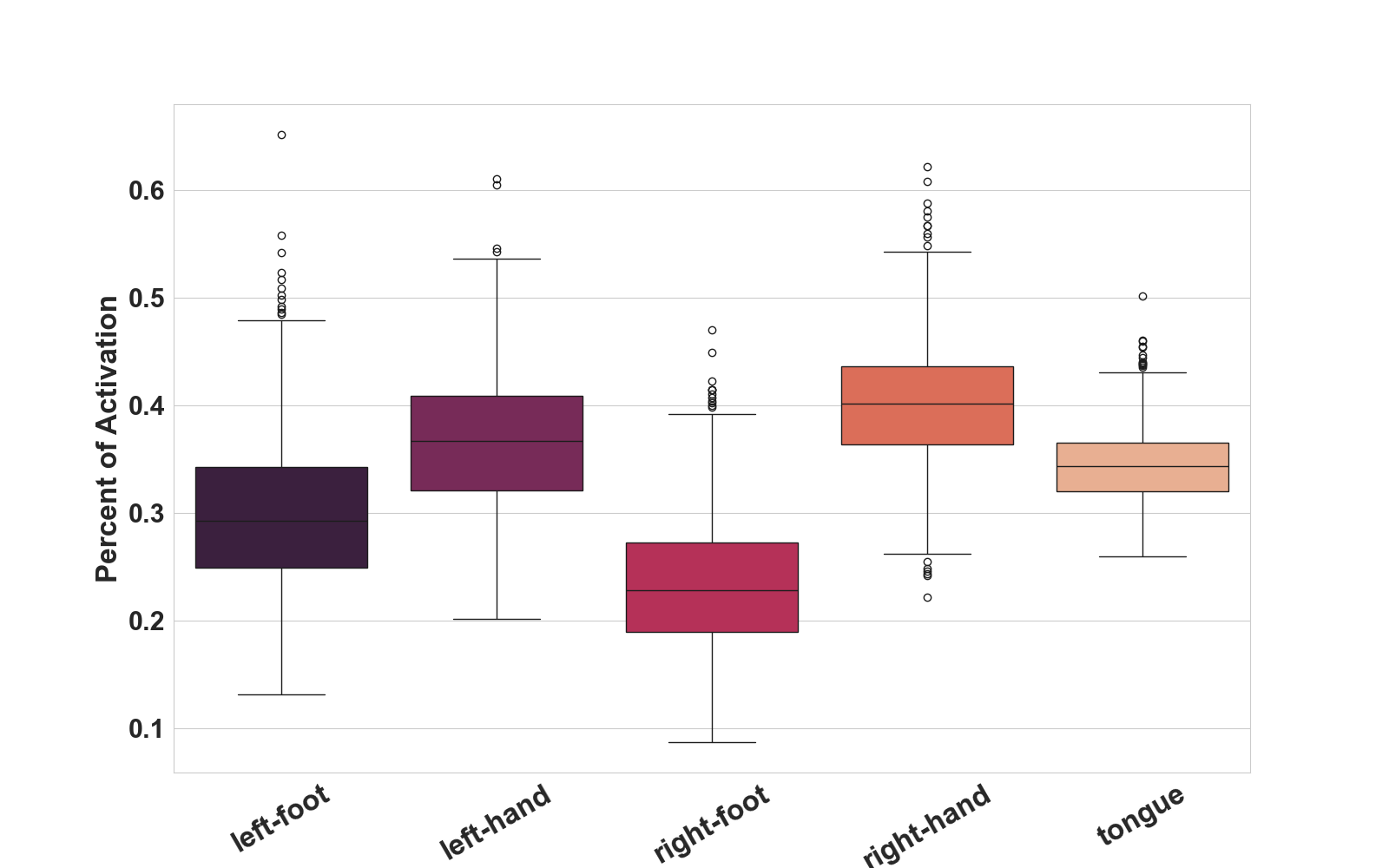

Supplement: Supplementary file 1 — Supplementary Materials [file 41597_2025_5247_MOESM1_ESM.zip › supplementary_materials/validation_FSL_8_24_1.png]

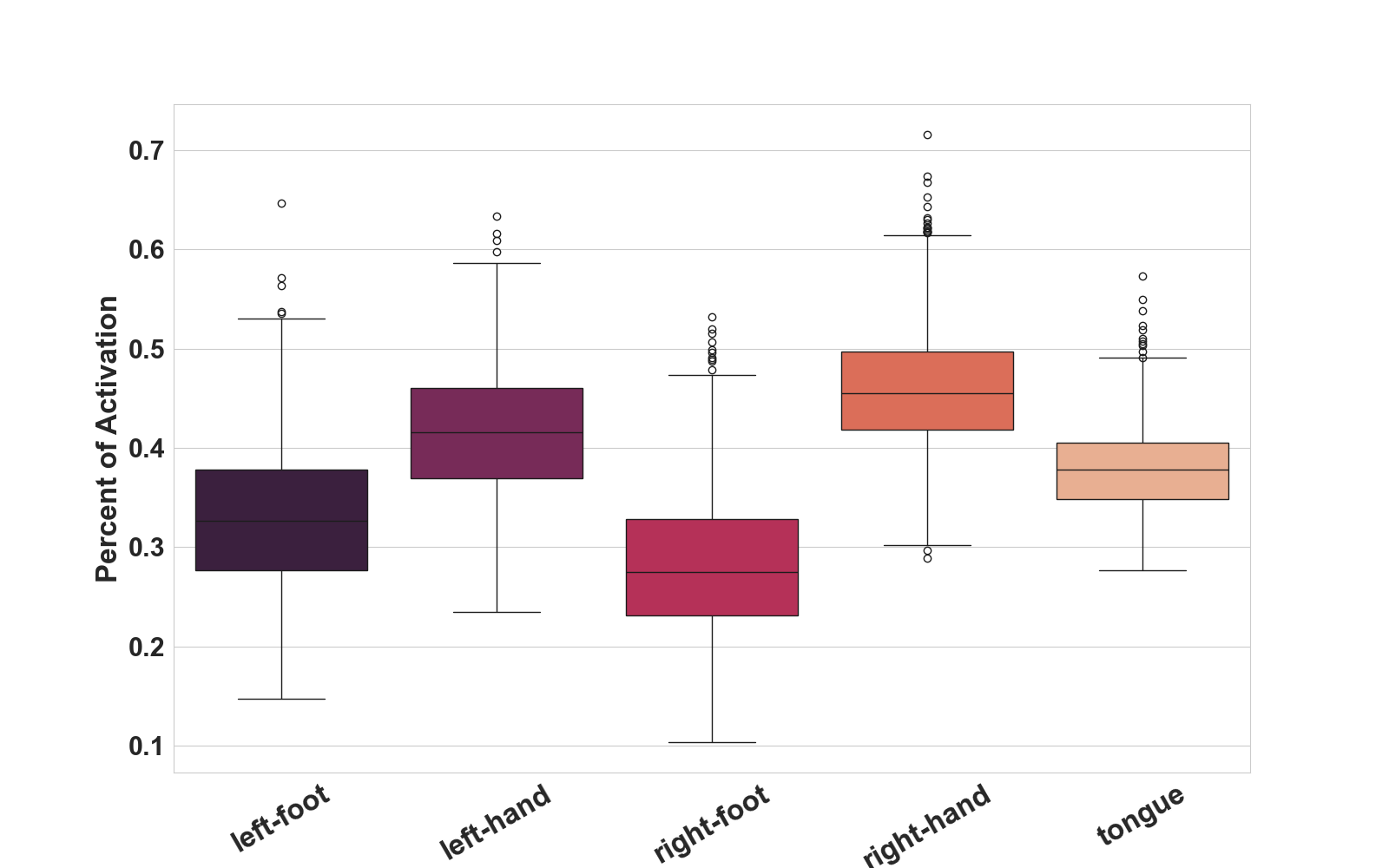

Supplement: Supplementary file 1 — Supplementary Materials [file 41597_2025_5247_MOESM1_ESM.zip › supplementary_materials/validation_FSL_8_24_0.png]

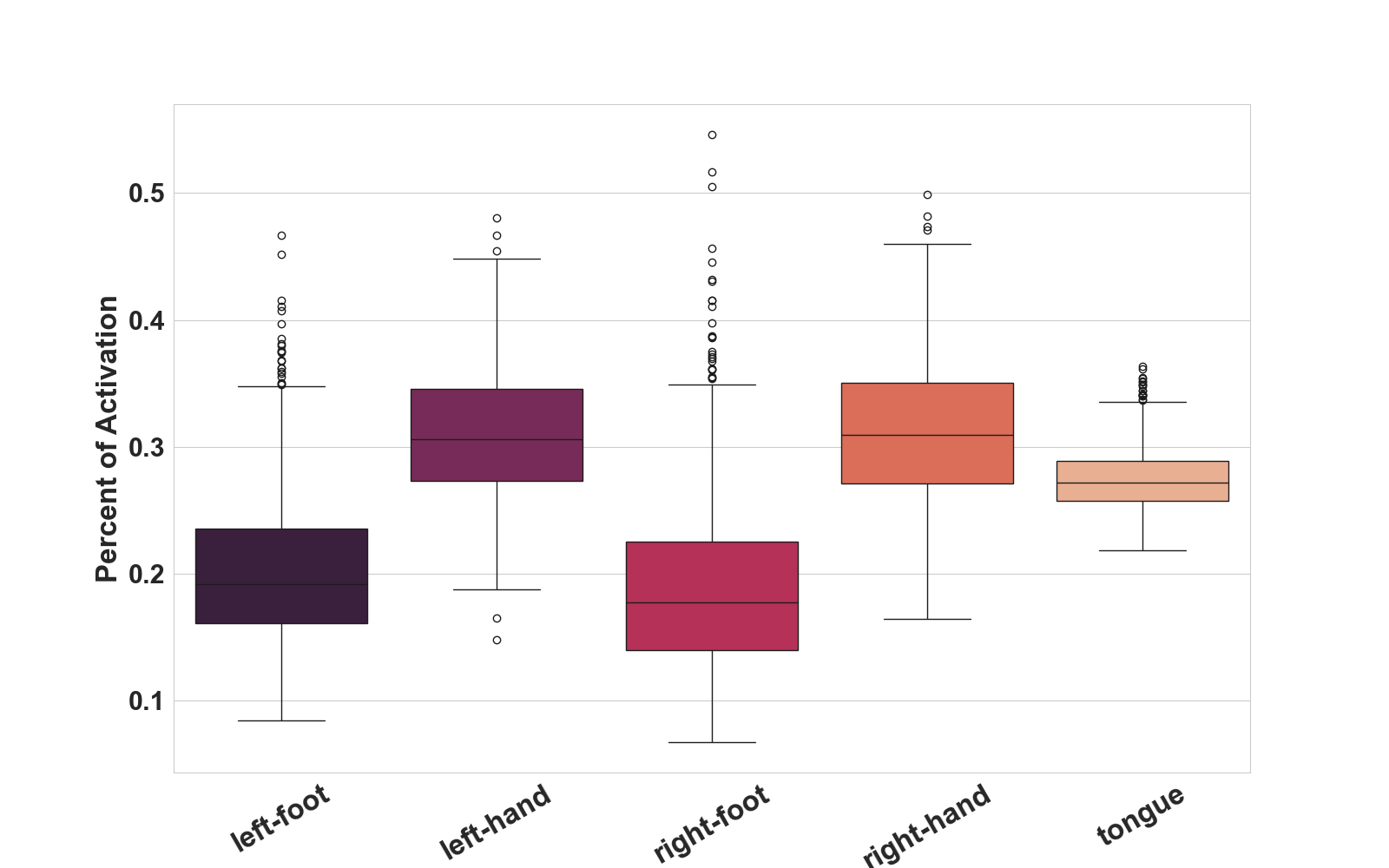

Supplement: Supplementary file 1 — Supplementary Materials [file 41597_2025_5247_MOESM1_ESM.zip › supplementary_materials/validation_SPM_8_6_1.png]

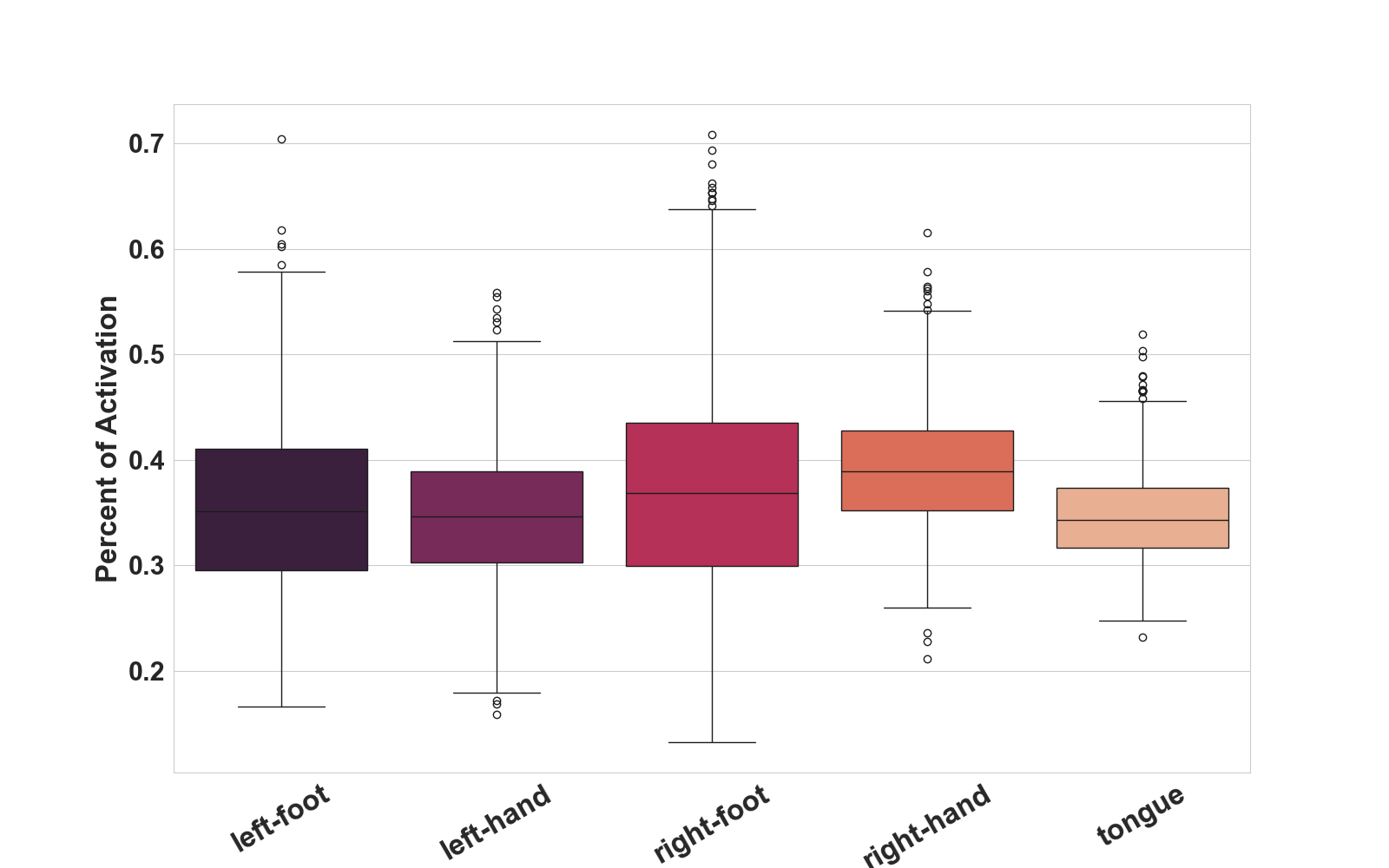

Supplement: Supplementary file 1 — Supplementary Materials [file 41597_2025_5247_MOESM1_ESM.zip › supplementary_materials/validation_FSL_5_0_0.png]

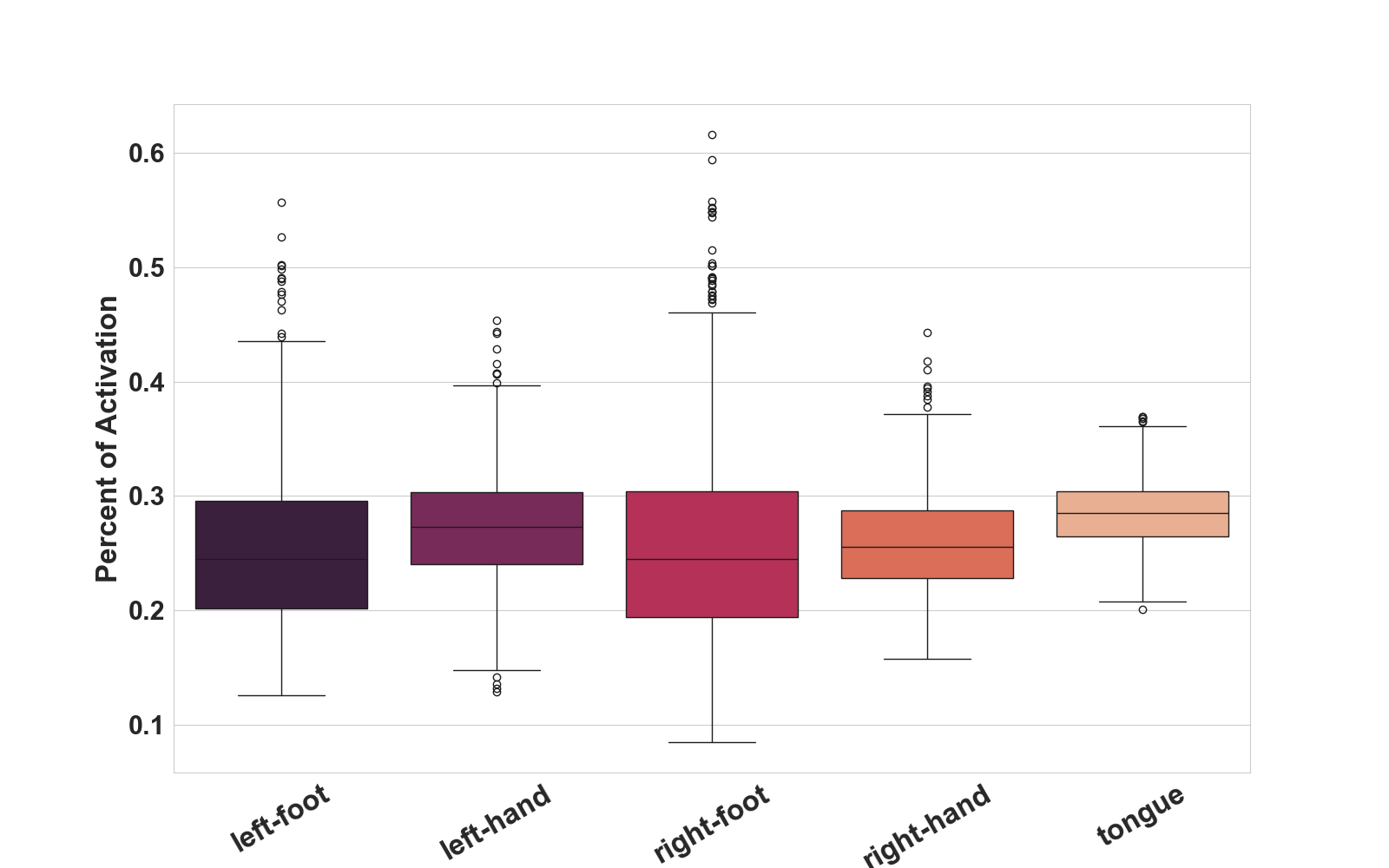

Supplement: Supplementary file 1 — Supplementary Materials [file 41597_2025_5247_MOESM1_ESM.zip › supplementary_materials/validation_FSL_5_0_1.png]

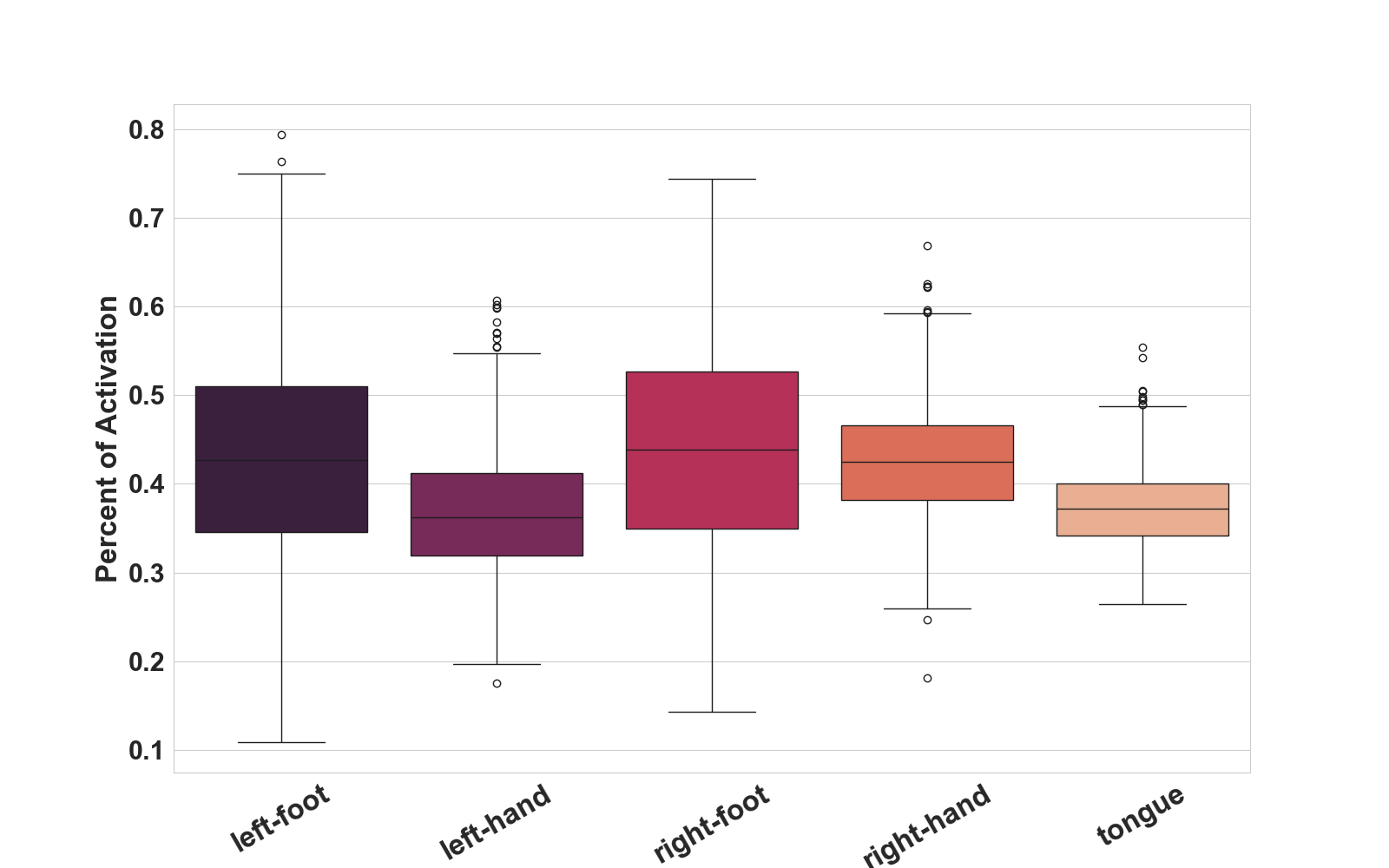

Supplement: Supplementary file 1 — Supplementary Materials [file 41597_2025_5247_MOESM1_ESM.zip › supplementary_materials/validation_SPM_8_0_0.png]

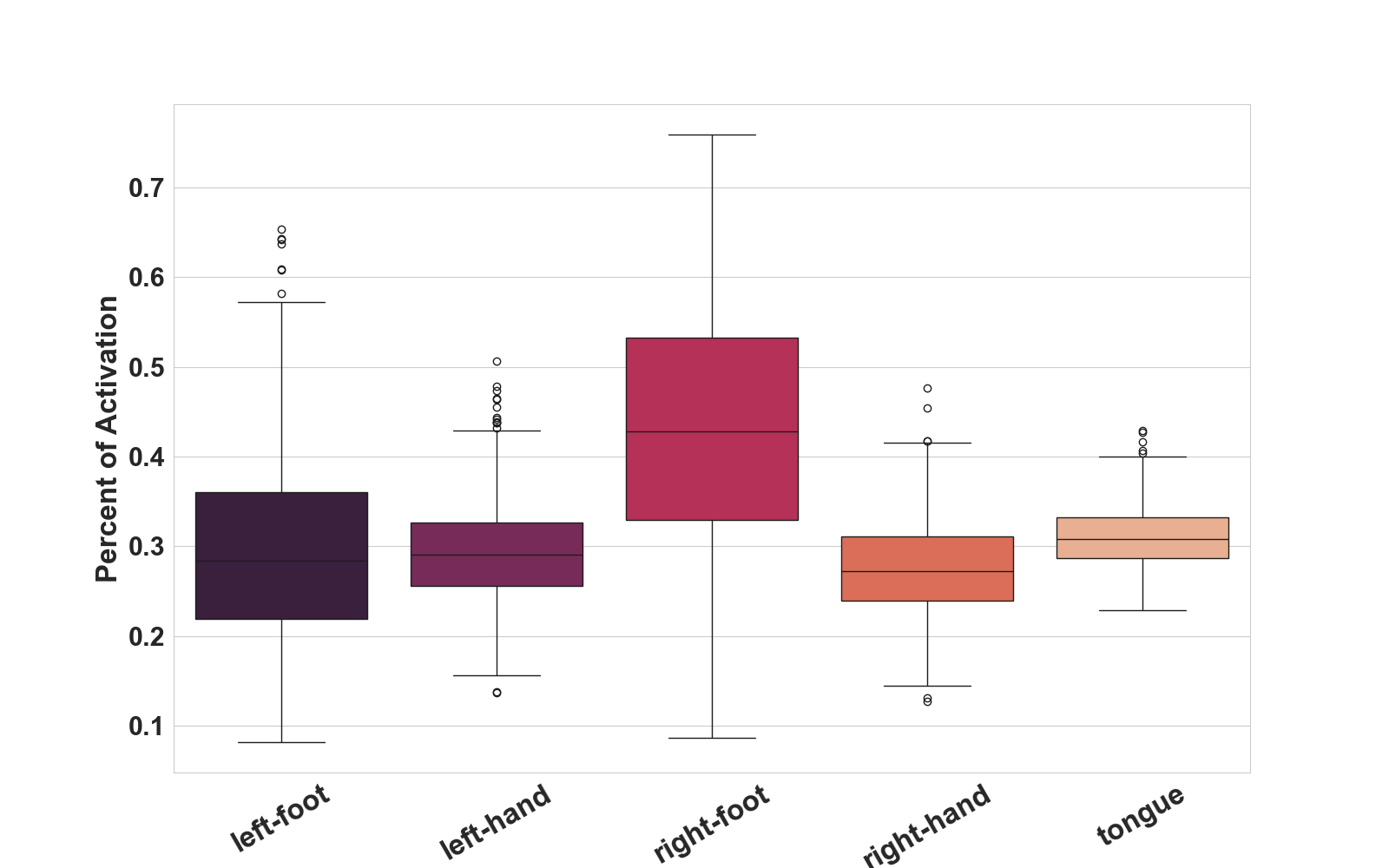

Supplement: Supplementary file 1 — Supplementary Materials [file 41597_2025_5247_MOESM1_ESM.zip › supplementary_materials/validation_SPM_8_0_1.png]

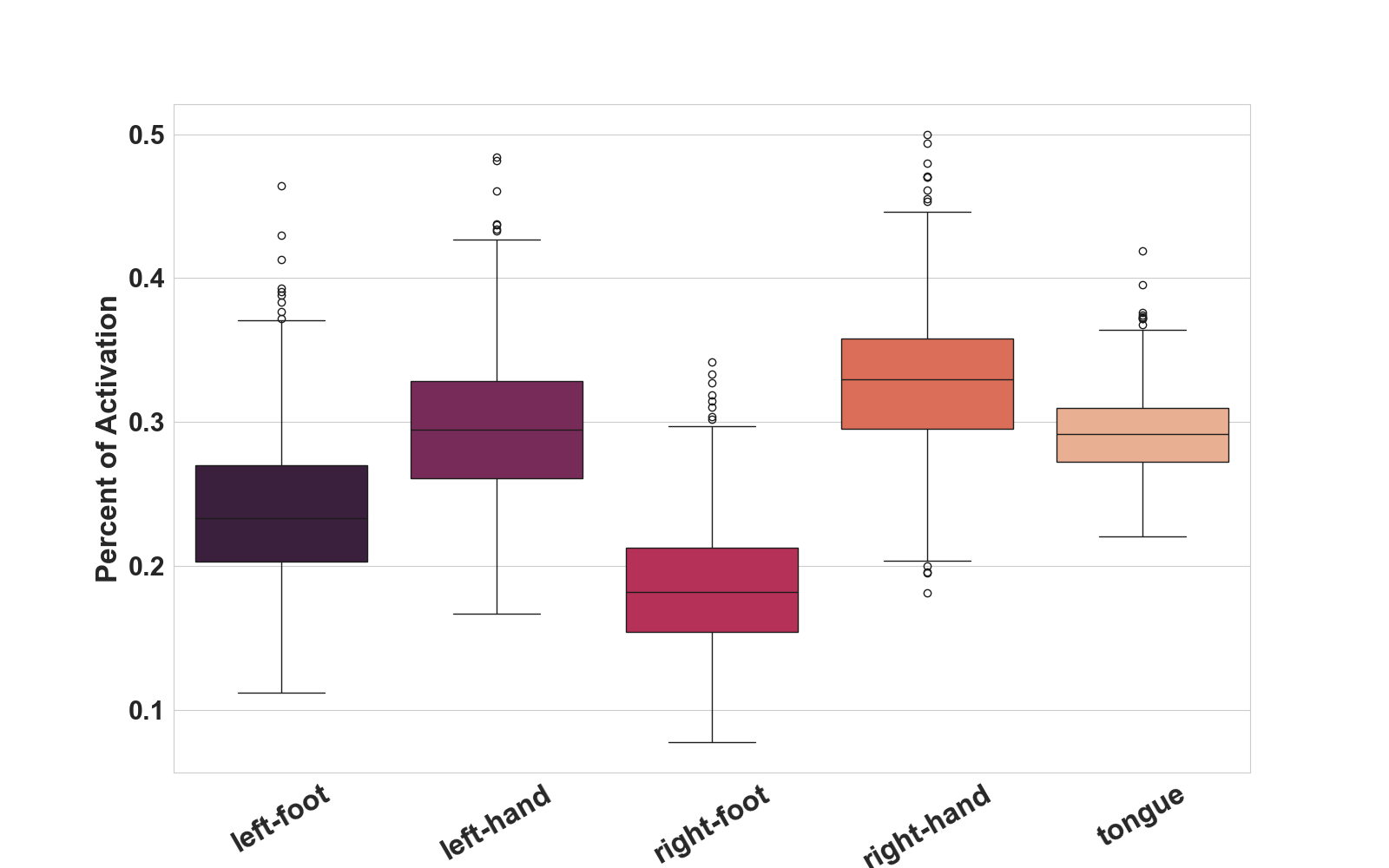

Supplement: Supplementary file 1 — Supplementary Materials [file 41597_2025_5247_MOESM1_ESM.zip › supplementary_materials/validation_FSL_5_24_1.png]

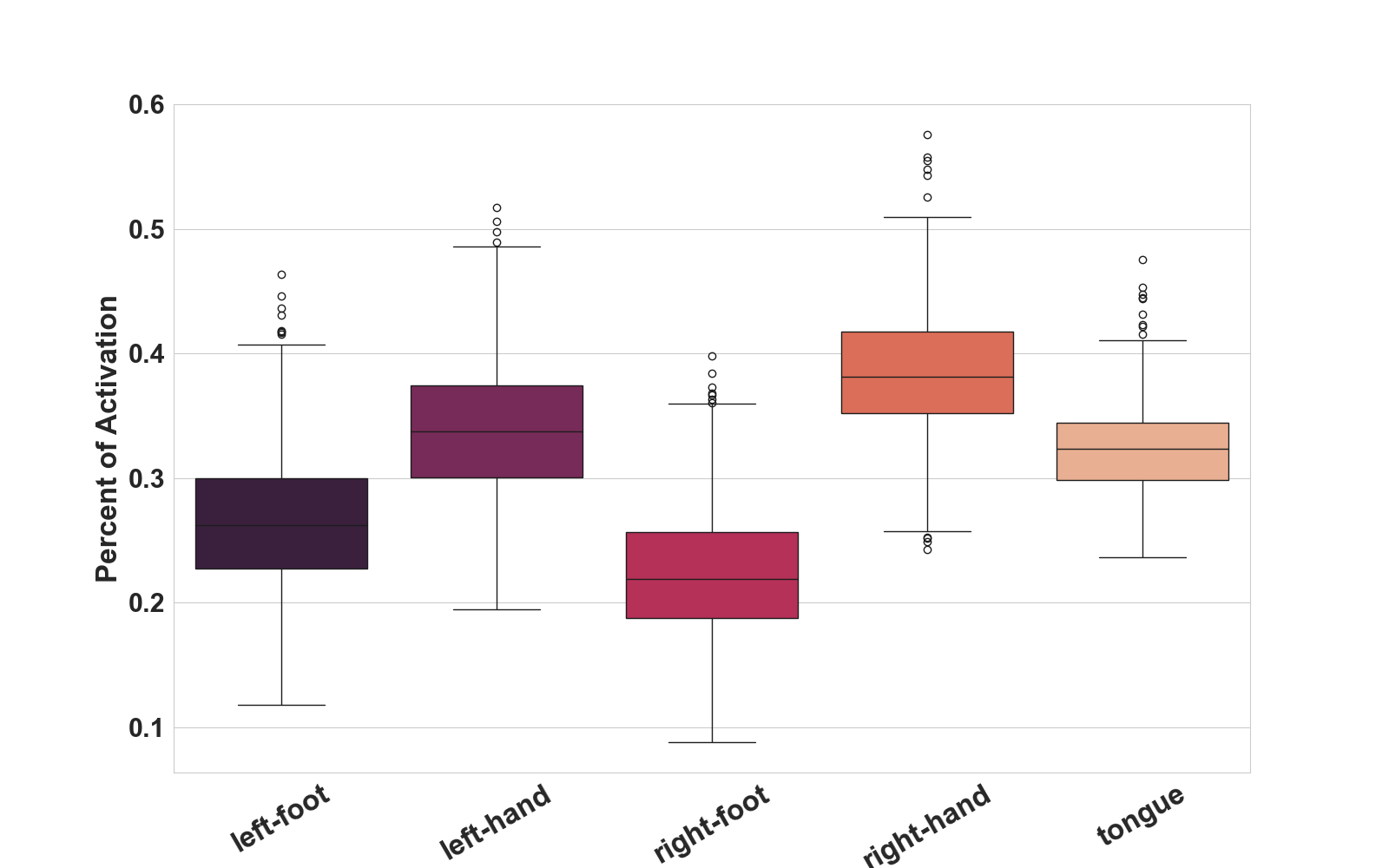

Supplement: Supplementary file 1 — Supplementary Materials [file 41597_2025_5247_MOESM1_ESM.zip › supplementary_materials/validation_FSL_5_24_0.png]

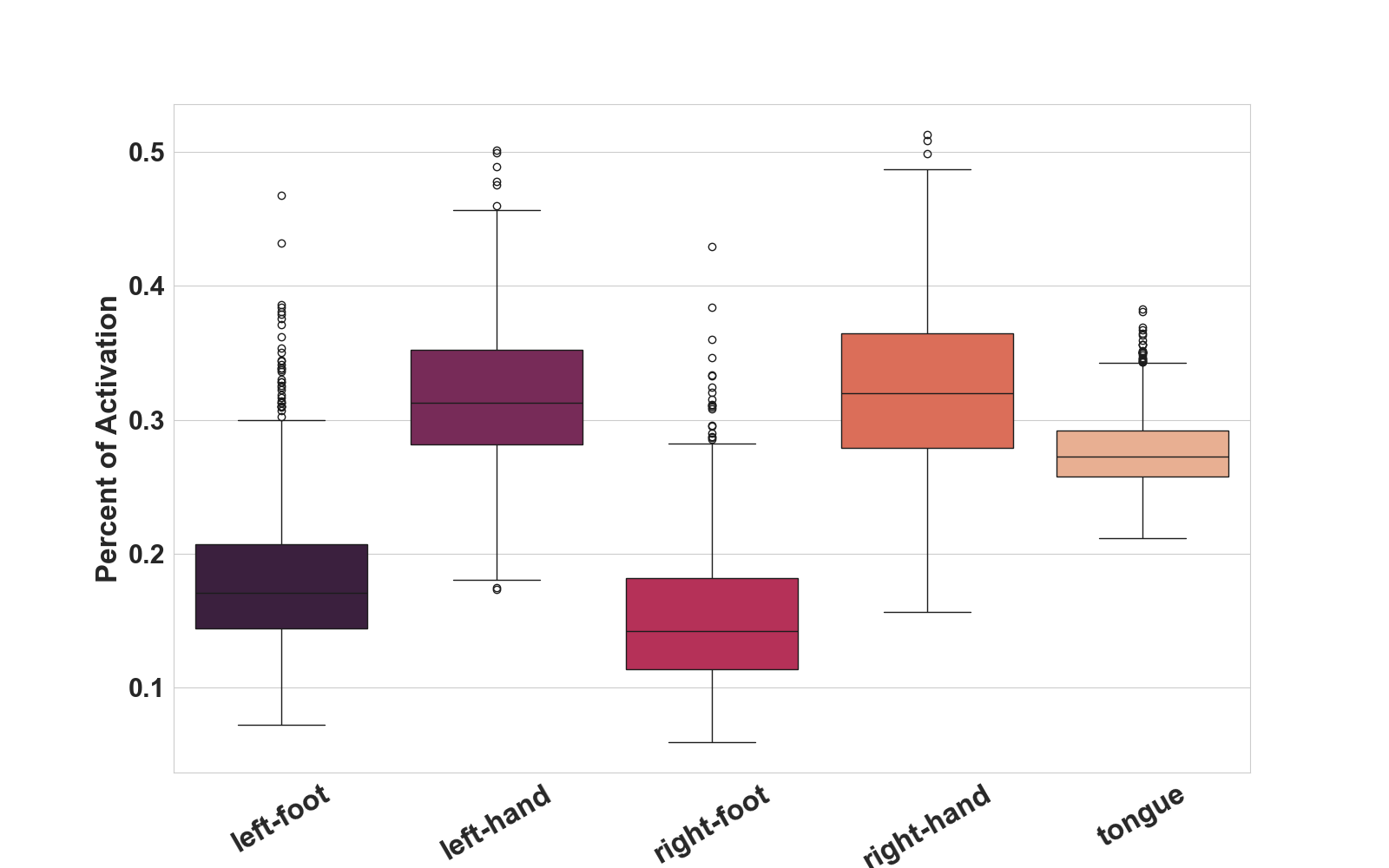

Supplement: Supplementary file 1 — Supplementary Materials [file 41597_2025_5247_MOESM1_ESM.zip › supplementary_materials/validation_SPM_8_24_1.png]

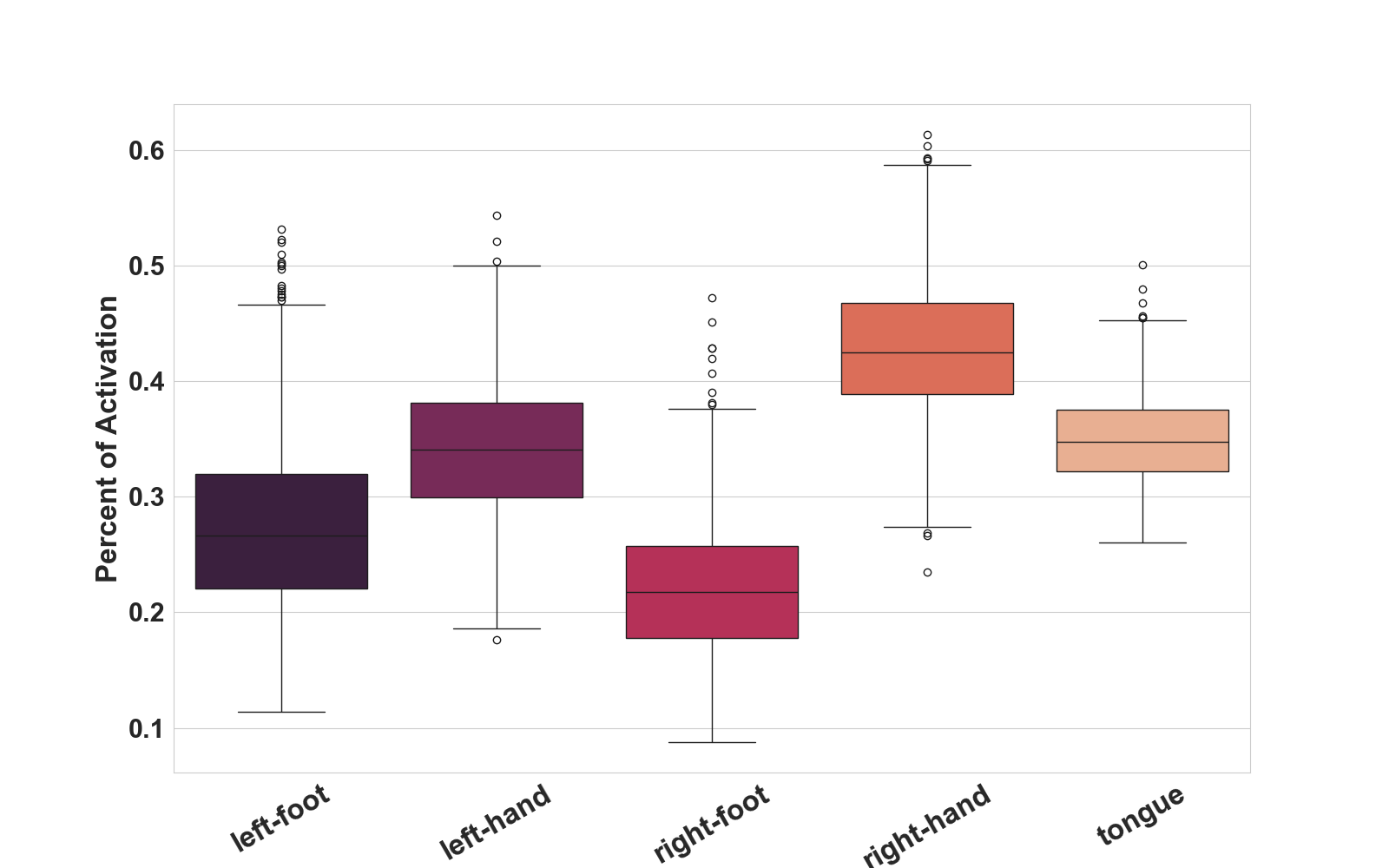

Supplement: Supplementary file 1 — Supplementary Materials [file 41597_2025_5247_MOESM1_ESM.zip › supplementary_materials/validation_SPM_8_24_0.png]

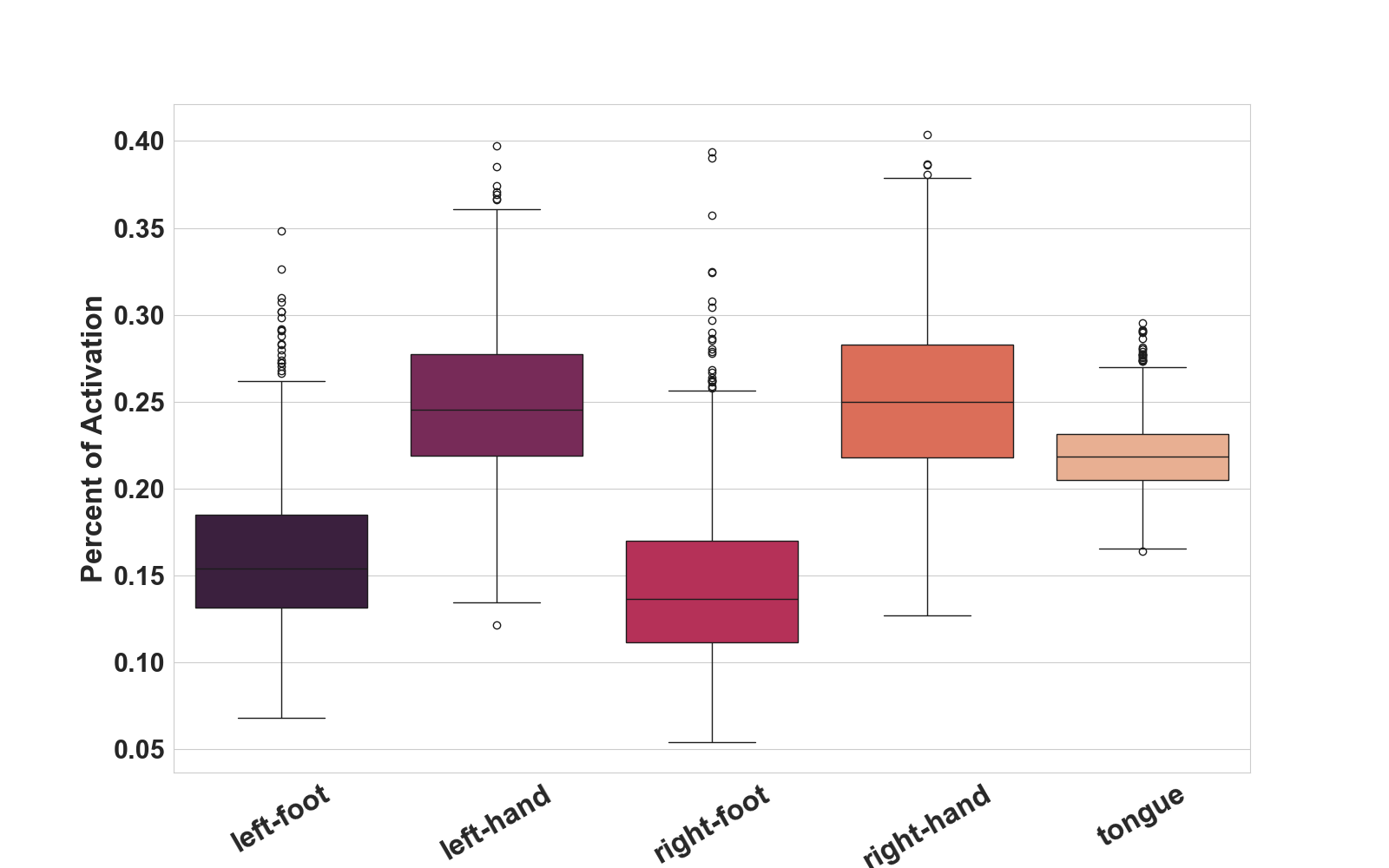

Supplement: Supplementary file 1 — Supplementary Materials [file 41597_2025_5247_MOESM1_ESM.zip › supplementary_materials/validation_SPM_5_6_1.png]

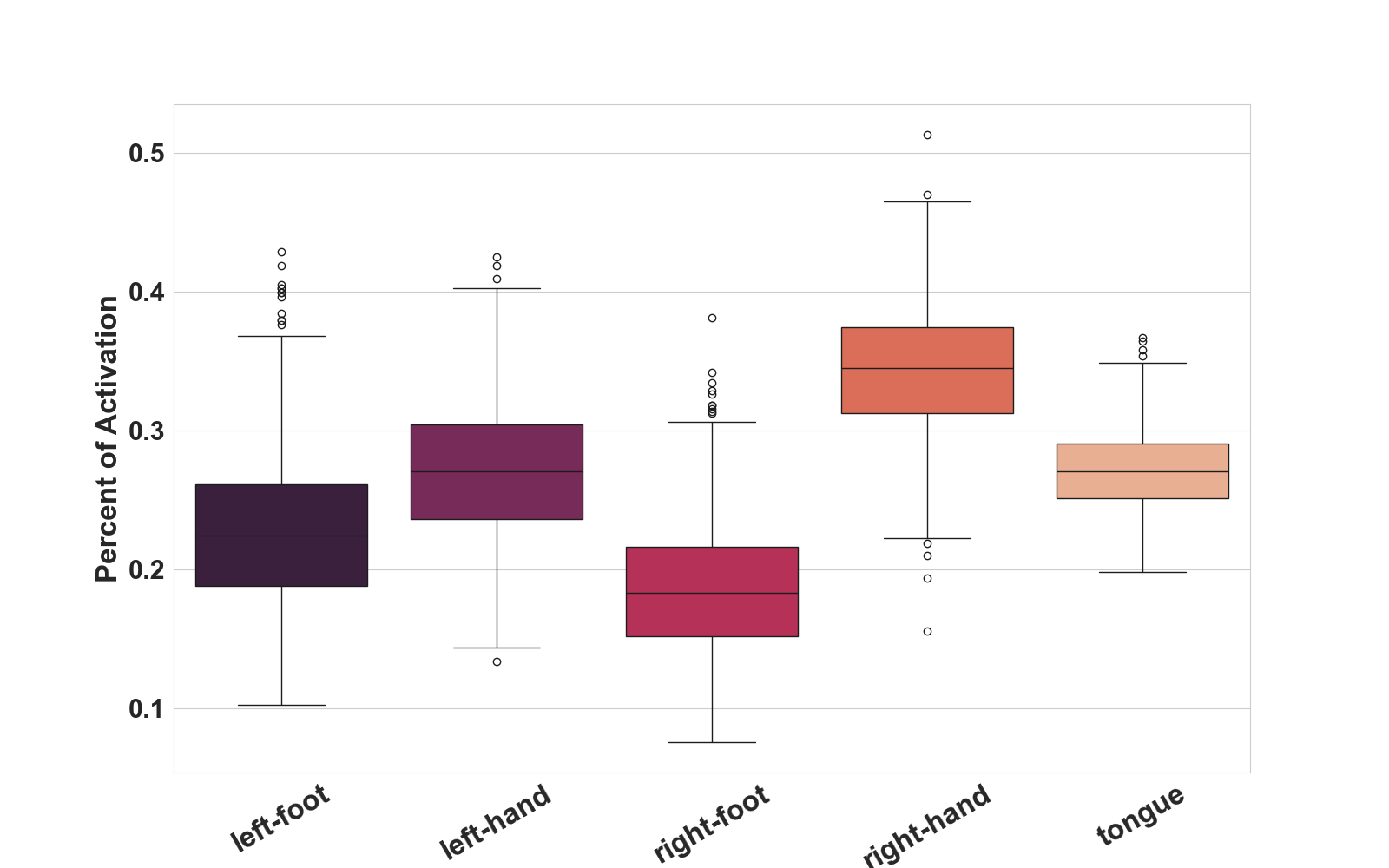

Supplement: Supplementary file 1 — Supplementary Materials [file 41597_2025_5247_MOESM1_ESM.zip › supplementary_materials/validation_SPM_5_6_0.png]

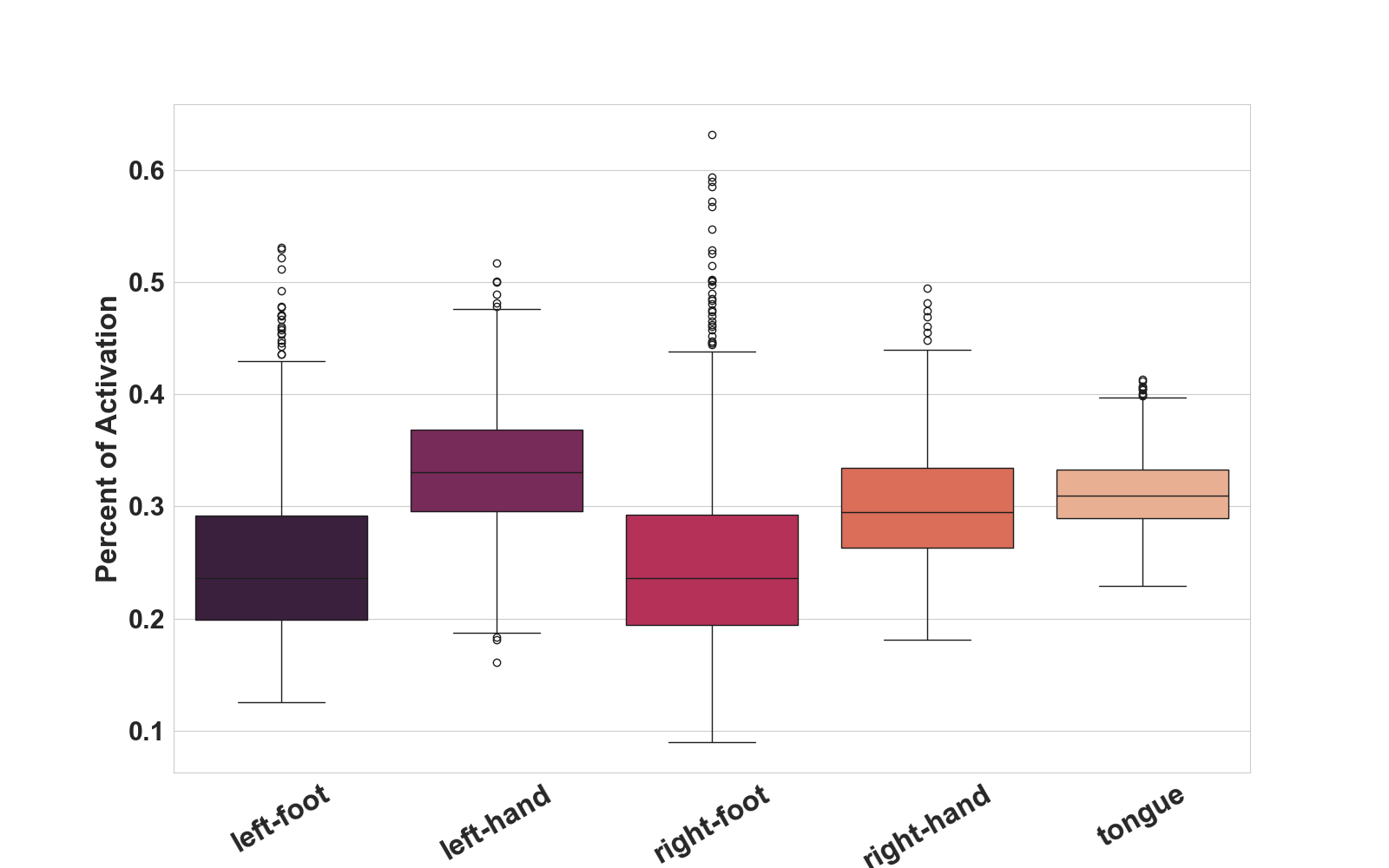

Supplement: Supplementary file 1 — Supplementary Materials [file 41597_2025_5247_MOESM1_ESM.zip › supplementary_materials/validation_FSL_8_6_1.png]

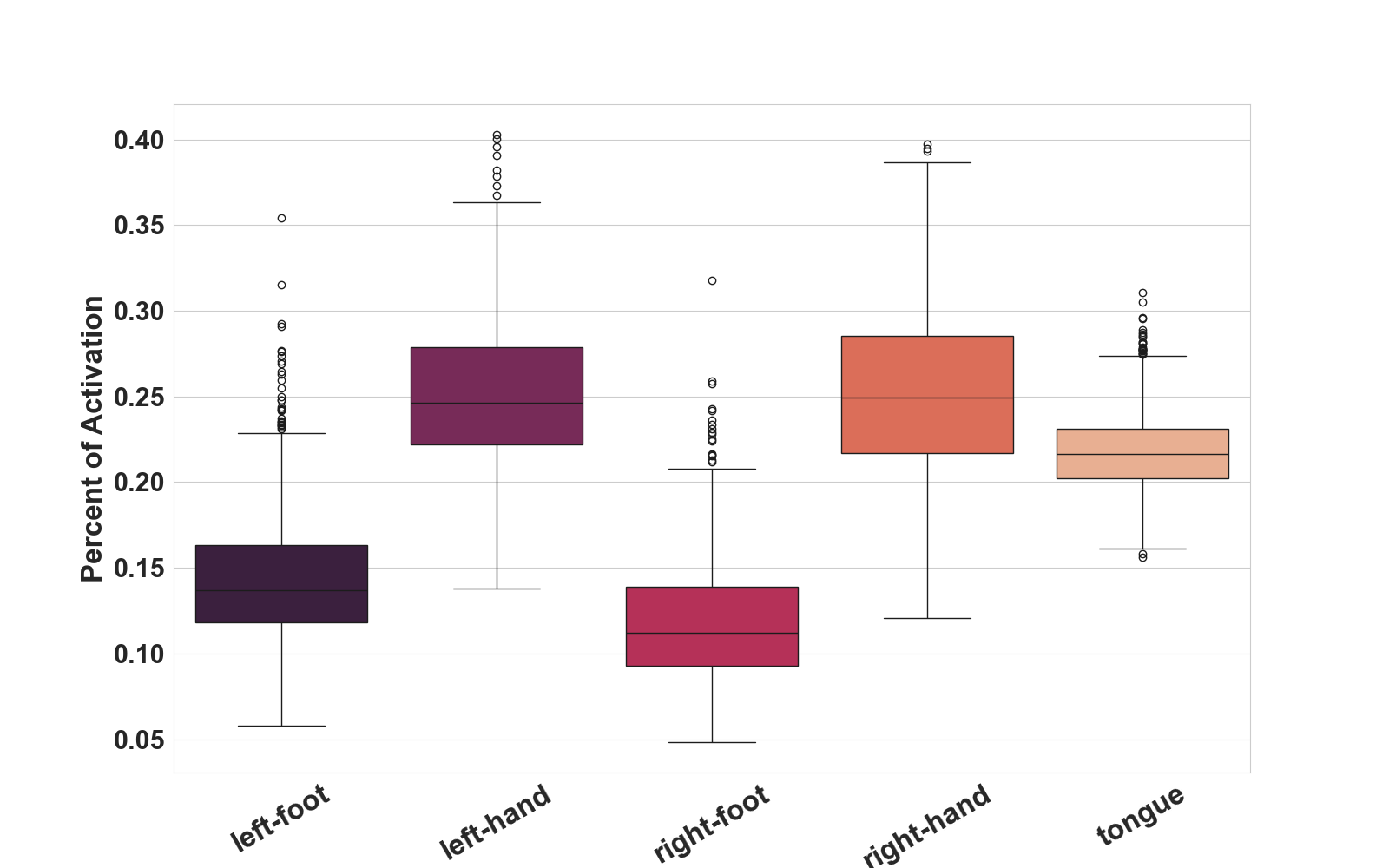

Supplement: Supplementary file 1 — Supplementary Materials [file 41597_2025_5247_MOESM1_ESM.zip › supplementary_materials/validation_SPM_5_24_1.png]

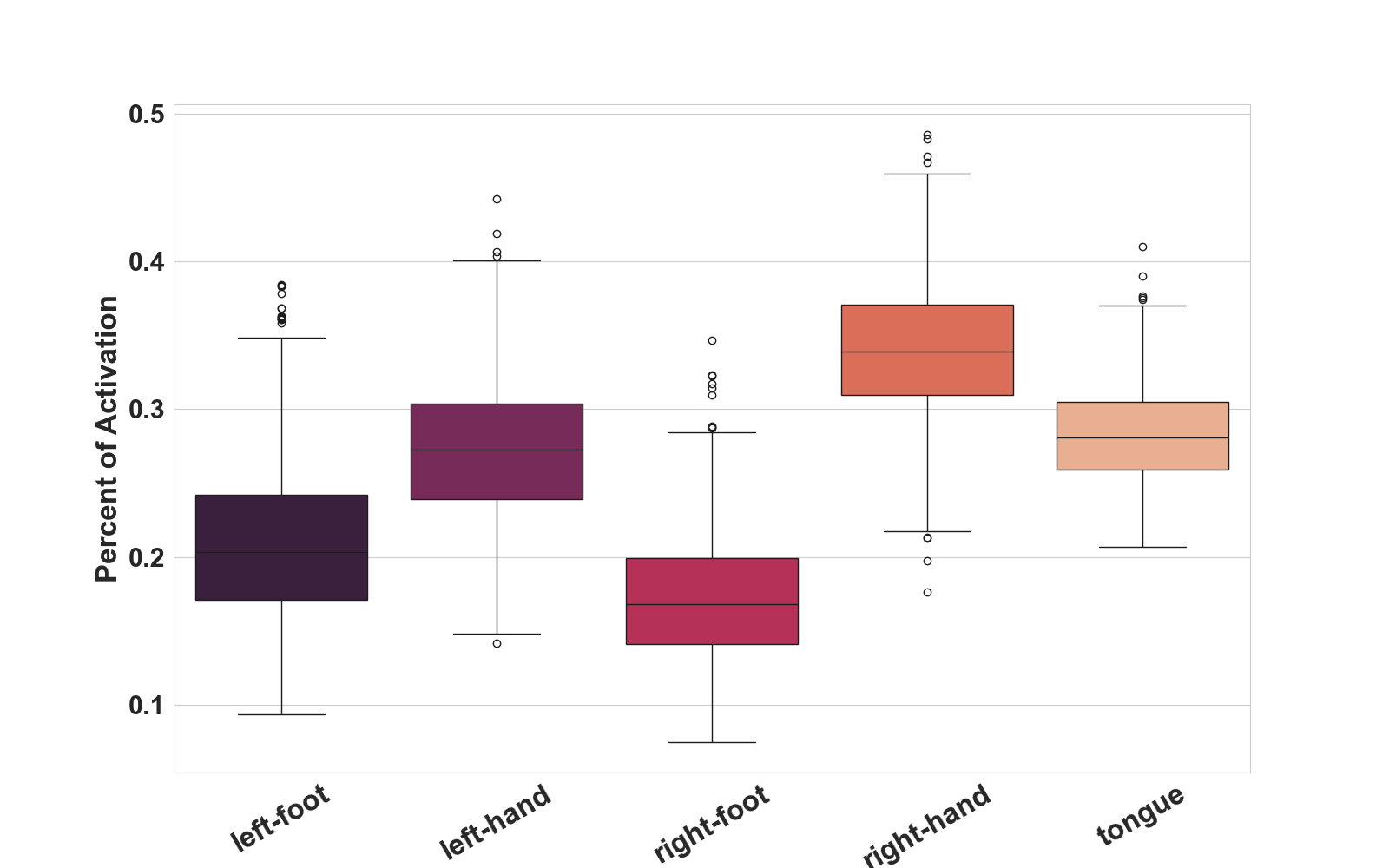

Supplement: Supplementary file 1 — Supplementary Materials [file 41597_2025_5247_MOESM1_ESM.zip › supplementary_materials/validation_SPM_5_24_0.png]

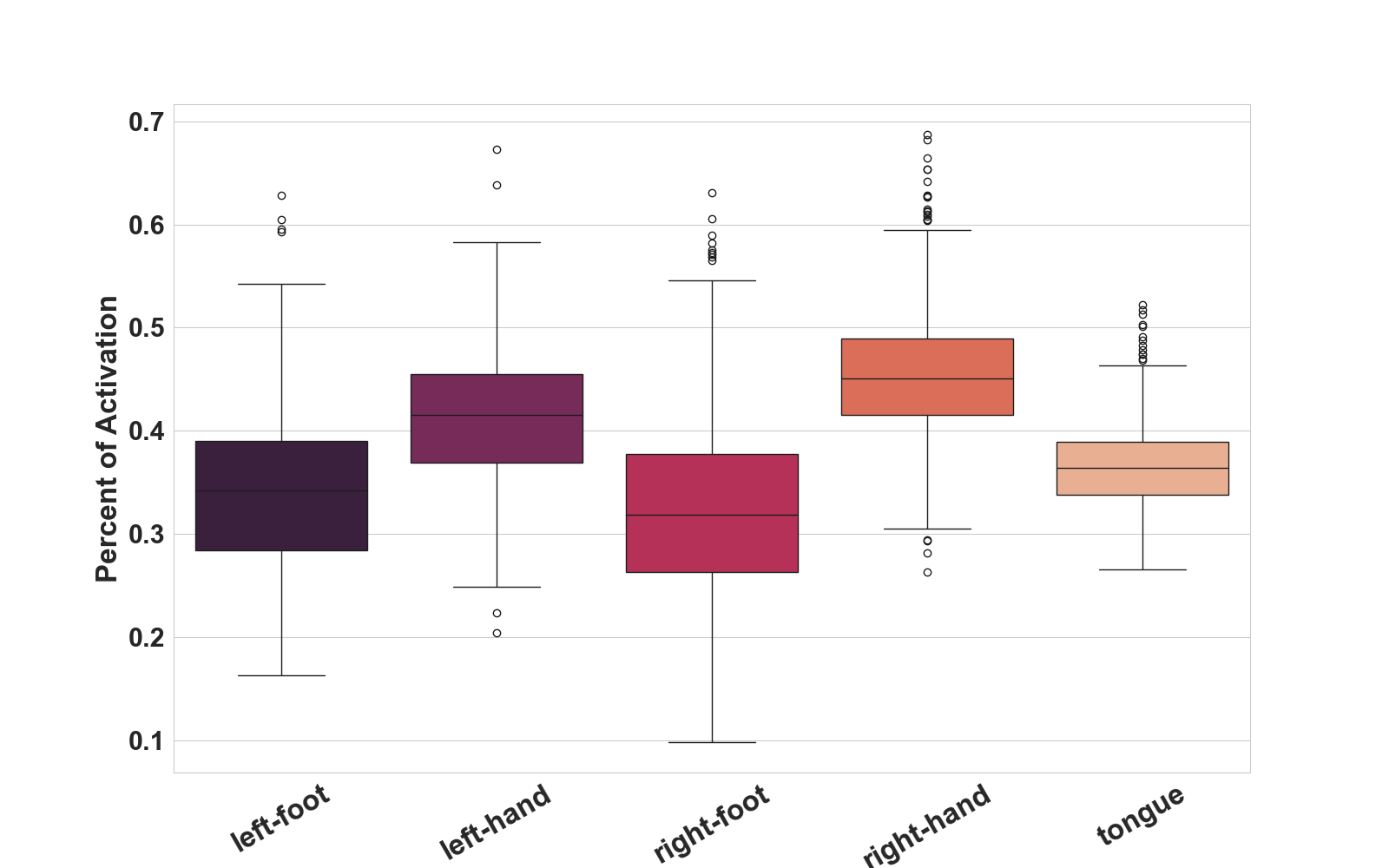

Supplement: Supplementary file 1 — Supplementary Materials [file 41597_2025_5247_MOESM1_ESM.zip › supplementary_materials/validation_FSL_8_6_0.png]

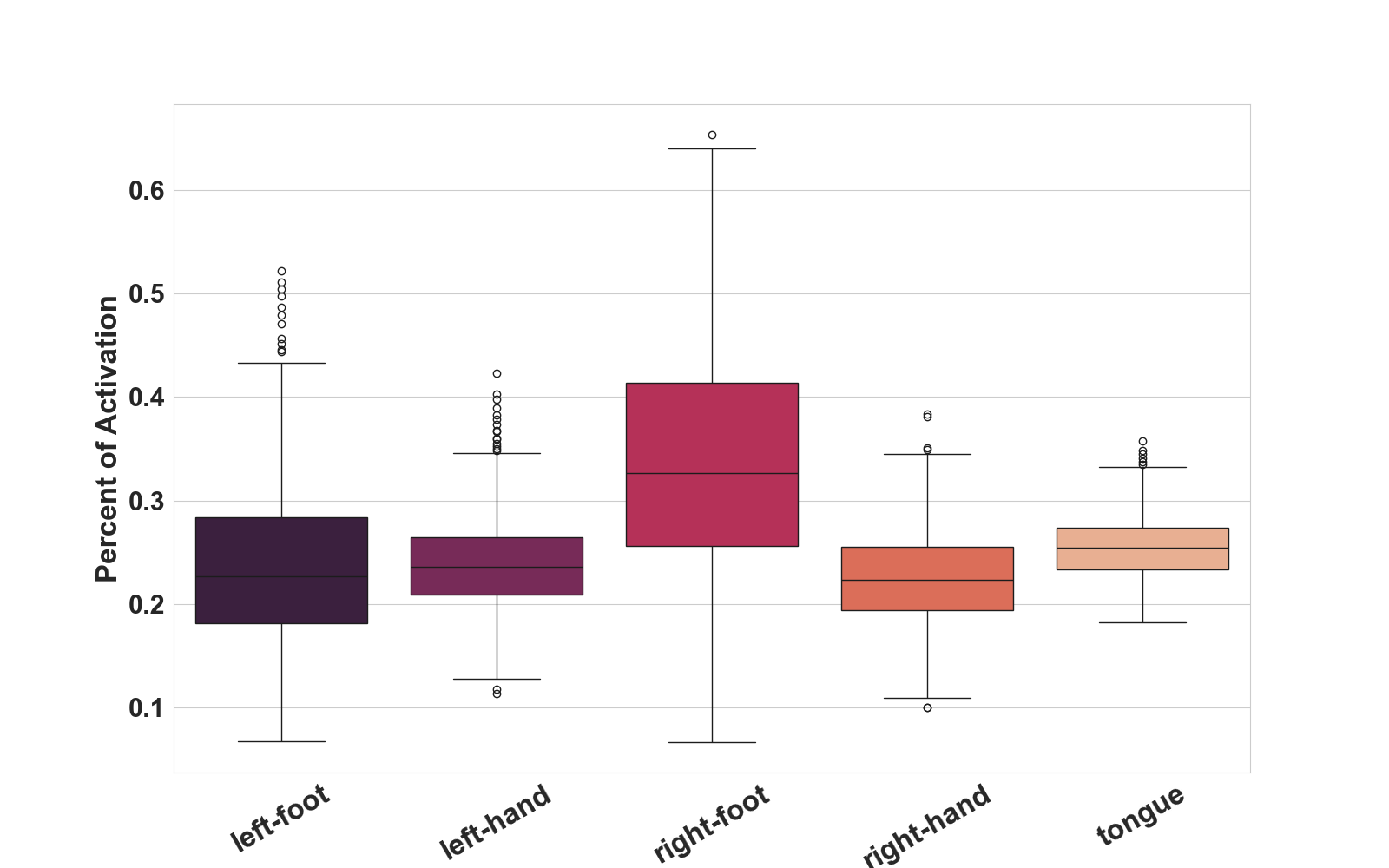

Supplement: Supplementary file 1 — Supplementary Materials [file 41597_2025_5247_MOESM1_ESM.zip › supplementary_materials/validation_SPM_5_0_1.png]

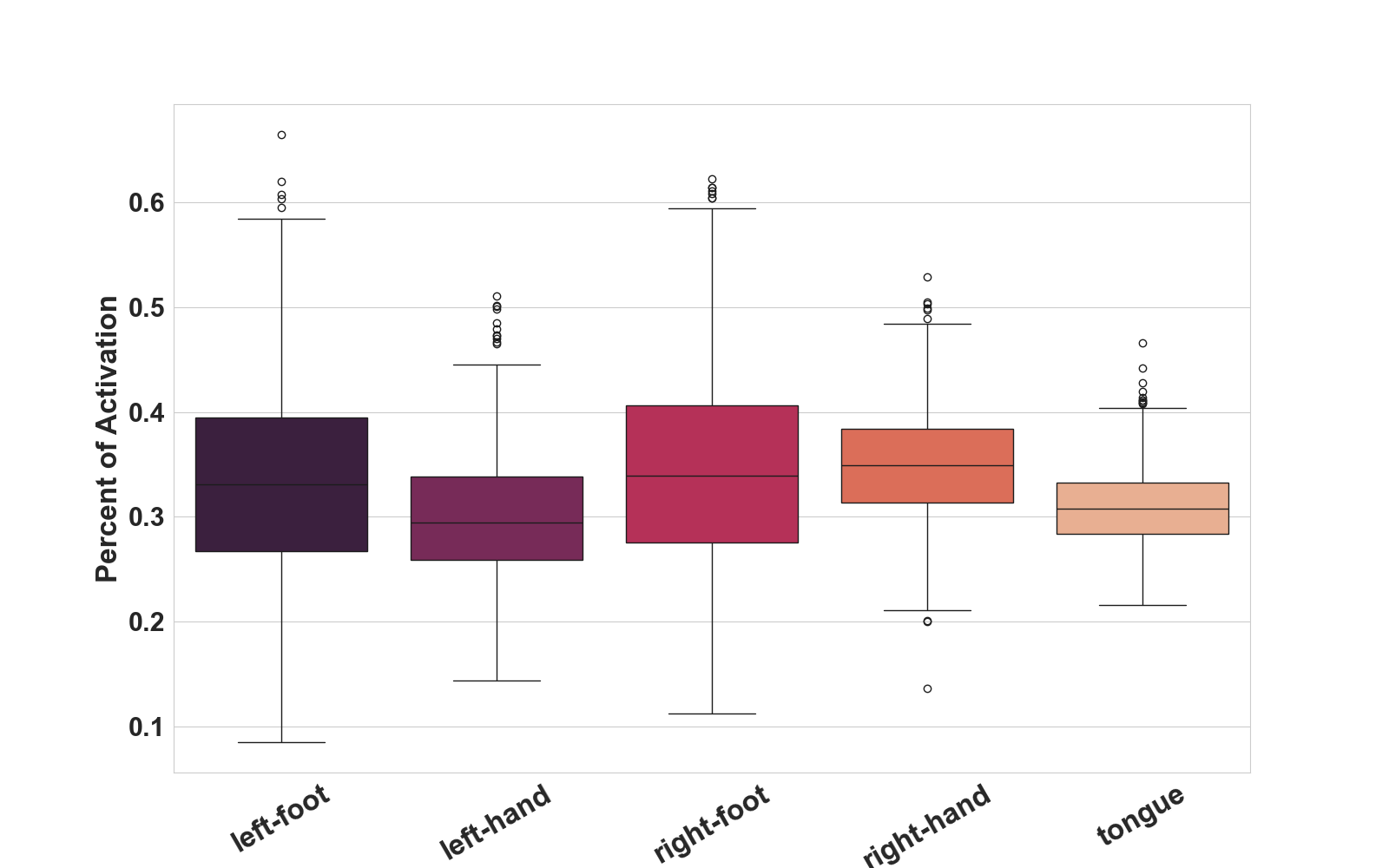

Supplement: Supplementary file 1 — Supplementary Materials [file 41597_2025_5247_MOESM1_ESM.zip › supplementary_materials/validation_SPM_5_0_0.png]

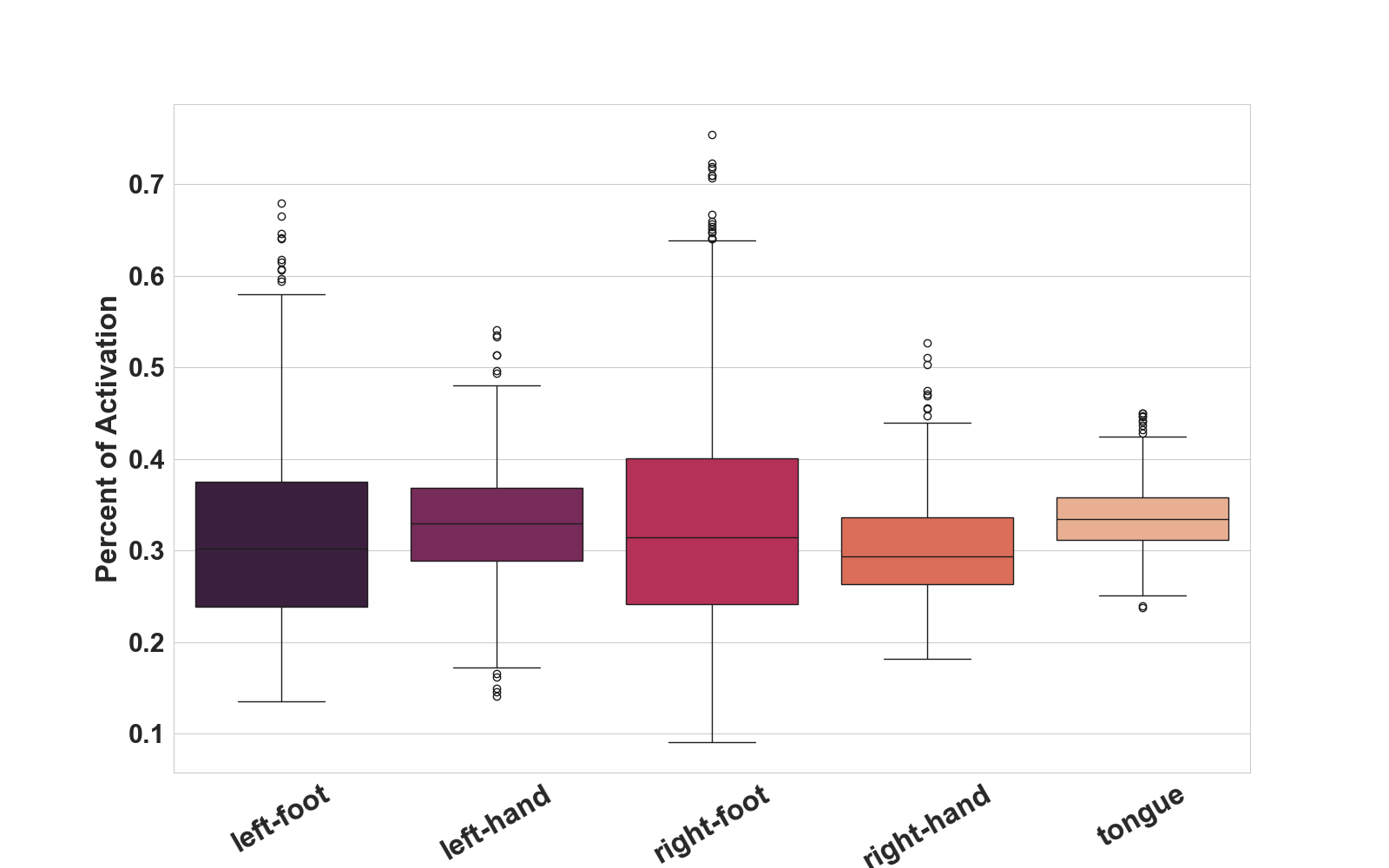

Supplement: Supplementary file 1 — Supplementary Materials [file 41597_2025_5247_MOESM1_ESM.zip › supplementary_materials/validation_FSL_8_0_1.png]

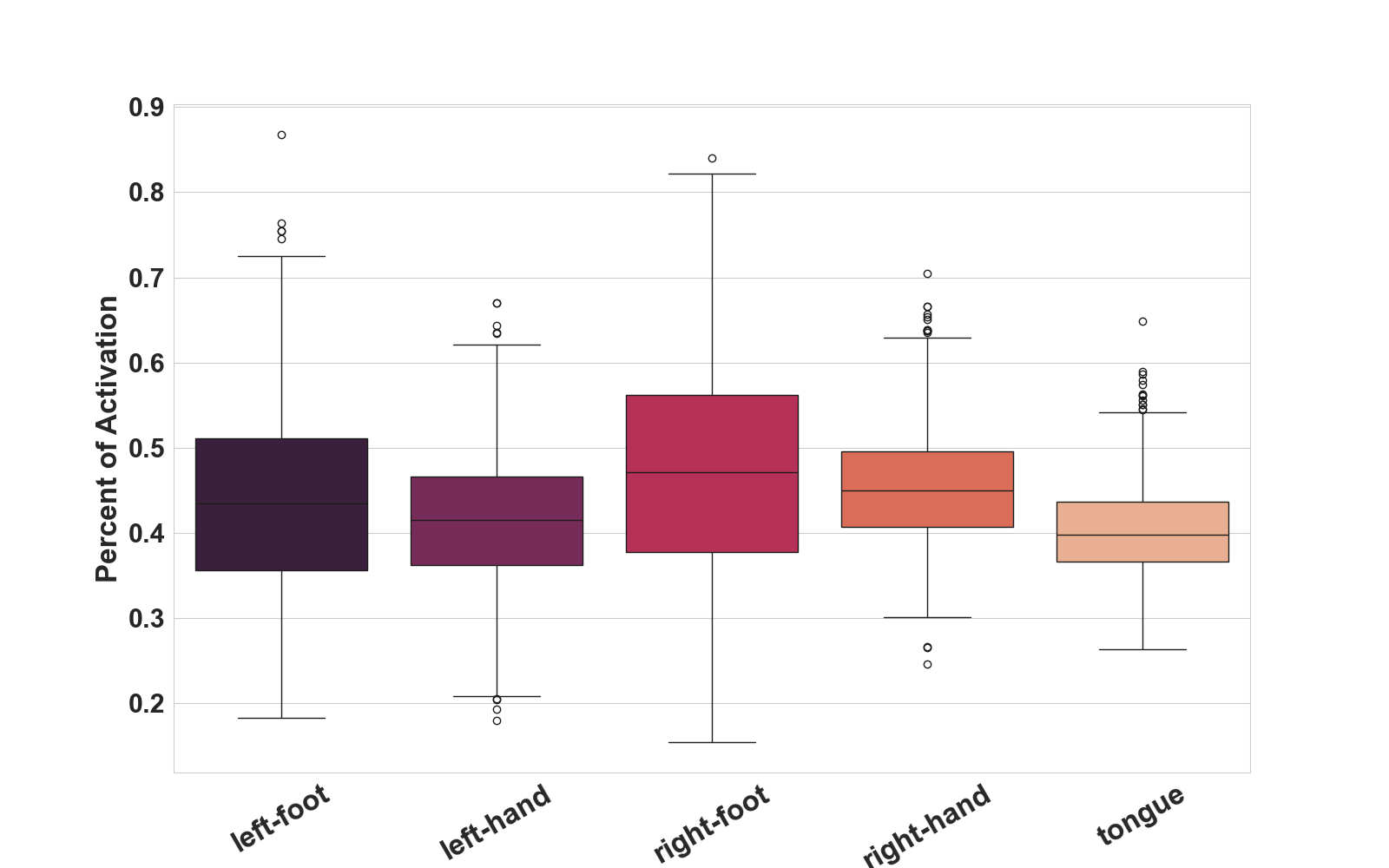

Supplement: Supplementary file 1 — Supplementary Materials [file 41597_2025_5247_MOESM1_ESM.zip › supplementary_materials/validation_FSL_8_0_0.png]
